# Racial Disparities and Preventive Measures to Renal Cell Carcinoma

**DOI:** 10.3390/ijerph15061089

**Published:** 2018-05-28

**Authors:** Jennifer N. Sims, Clement G. Yedjou, Daniel Abugri, Marinelle Payton, Timothy Turner, Lucio Miele, Paul B. Tchounwou

**Affiliations:** 1 Department of Behavioral and Environmental Health, School of Public Health, Jackson State University, 350 W. Woodrow Wilson Dr., P.O. Box 17038, Jackson, MS 39217, USA; jennifer.n.sims@jsums.edu (J.N.S.); marinelle.payton@jsums.edu (M.P.); 2 Department of Biology, College of Science, Engineering and Technology, Jackson State University, 1400 Lynch St., Jackson, MS 39217, USA; timothy.turner@jsums.edu; 3 Natural Chemotherapeutics Research Laboratory, RCMI Center for Environmental Health, Jackson State University, 1400 Lynch St., Jackson, MS 39217, USA; 4 Department of Chemistry and Department of Biology, Laboratory of Ethno-Medicine, Parasitology and Drug Discovery, College of Arts and Science, Tuskegee University, 1200 Old Montgomery Road, Tuskegee, AL 36088, USA; dabugri@tuskegee.edu; 5 Department of Genetics, Louisiana State University, Health Sciences Center, School of Medicine, 533 Bolivar St., Room 657, New Orleans, LA 70112, USA; lmiele@lsuhsc.edu

**Keywords:** Renal cell carcinoma, incidence, mortality, risk factors, Black, White, prevention

## Abstract

Kidney cancer ranks among the top 10 cancers in the United States. Although it affects both male and female populations, it is more common in males. The prevalence rate of renal cell carcinoma (RCC), which represents about 85% of kidney cancers, has been increasing gradually in many developed countries. Family history has been considered as one of the most relevant risk factors for kidney cancer, although most forms of an inherited predisposition for RCC only account for less than four percent. Lifestyle and other factors such as occupational exposure, high blood pressure, poor diet, and heavy cigarette smoking are highly associated with its incidence and mortality rates. In the United States, White populations have the lowest prevalence of RCC compared to other ethnic groups, while Black Americans suffer disproportionally from the adverse effects of RCC. Hence, this review article aims at identifying the major risk factors associated with RCC and highlighting the new therapeutic approaches for its control/prevention. To achieve this specific aim, articles in peer-reviewed journals with a primary focus on risk factors related to kidney cancer and on strategies to reduce RCC were identified. The review was systematically conducted by searching the databases of MEDLINE, PUBMED Central, and Google Scholar libraries for original articles. From the search, we found that the incidence and mortality rates of RCC are strongly associated with four main risk factors, including family history (genetics), lifestyle (poor diet, cigarette smoking, excess alcohol drinking), environment (community where people live), and occupation (place where people work). In addition, unequal access to improvement in RCC cancer treatment, limited access to screening and diagnosis, and limited access to kidney transplant significantly contribute to the difference observed in survival rate between African Americans and Caucasians. There is also scientific evidence suggesting that some physicians contribute to racial disparities when performing kidney transplant among minority populations. New therapeutic measures should be taken to prevent or reduce RCC, especially among African Americans, the most vulnerable population group.

## 1. Introduction

Renal cell carcinoma (RCC) is a group of malignancies that arises from the epithelium of the renal tubules. In 2004, RCC was classified by the World Health Organization as consisting of distinct subtypes of adult renal cancers, which include clear cell, papillary, and chromophobe tumors, representing approximately 70%, 10%–15%, and 5%, respectively [1]. Many rare cases of RCC subtypes include: (a) carcinoma of the collecting ducts of Bellini; (b) Xp11.2 translocation carcinoma; (c) multilocular cell renal carcinoma; (d) renal medullary carcinoma; (e) carcinoma associated with neuroblastoma; (f) mucinous tubular and spindle cell carcinoma and unclassified RCC [1]. Another rare case of RCC associated with aggressive behavior and poor prognosis that may happen in any subtype is sarcomatoid [1]. In 2018 alone, it is expected that approximately 63,340 new people will develop renal cancer in the United States and about 14,970 cancer-related deaths (10,010 in men and 4960 in women) will occur [2]. RCC affects both males and females, but it is more common in males compared to females [3]. During the past two decades, the incidence rate of RCC has significantly declined among Caucasians but has rapidly increased among African Americans [4]. There is an immense quality improvement of RCC in the literature among Caucasians. However, there are limited studies that have addressed the prevention of RCC among underrepresented minority groups. Also, very few studies have focused on how to reduce RCC racial health disparities. Therefore, this review article aims to identify major risk factors associated with RCC and to highlight new therapeutic approaches for the prevention of RCC.

## 2. Approaches

We performed bibliographical searches in Google Scholar (https://scholar.google.com), cancer journals, and Researchgates. In addition, articles that were not visible at these search tools were further searched and identified through MEDLINE and PUBMED Central links with the primary focus on epidemiologic research studies that provide new insights regarding the main factors that contribute to renal cell carcinoma development and the therapeutic approaches that aim at preventing this disease.

## 3. Results and Discussions

We found several peer-reviewed papers that addressed and explained the differences in renal cell carcinoma (RCC) incidence and mortality rates between African Americans and Caucasians. The summary results and discussions of this paper give an update of the overview of RCC, racial/ethnic disparities, major risk factors of RCC, and new therapeutic approaches that aim at preventing RCC. In our results and discussion sections, we did not summarize all the major risk factors and therapeutic approaches investigated in each study. However, we highlighted their main findings in the present article. These are discussed in more detail in subsequent sections below.

### 3.1. Racial Disparities in Renal Cell Carcinoma

According to the CDC, in the United States in 2014, among men, Black men are the most likely to get kidney and renal pelvis cancers (24.7 per 100,000), followed by White men (22.0 per 100,000). Among women, African American women are the most like to get kidney and renal pelvis cancers (12.4 per 100,000), followed by Hispanic women (11.9 per 100,000) [5]. Research has shown that race influences the distribution of histologic subtypes, with African American patients having increased frequency of papillary kidney cancer [6,7]. African Americans generally present at an earlier disease stage [8,9,10,11]; however, survival rates are worse [10,12]. Studies suggest that the distribution of RCC histological subtypes is not equivalent in different racial groups [6,8]. It has been shown that clear cell RCC is more common in Caucasian populations and papillary RCC is more common in people of African or Afro-Caribbean descent [6]. A disparity in relative survival still persists between African Americans and Caucasians patients with kidney cancer even when controlling for treatment and other prognostic factors including stage, tumor size, and grade [12]. Several Surveillance, Epidemiology, and End Results (SEER) analyses and state-wide cancer registry data revealed that African Americas with RCC consistently had higher all-cause mortality rates than Whites in the general population [10,11,12,13]. The cause of this disparity in mortality is unknown. Some studies attribute this disparity to racial differences in access to health care and treatment [10,11,12,13,14,15], differences in quality of care received [12,13], patients’ attitudes toward and beliefs in treatment decisions [12,13], comorbid conditions, and stressful life events associated with socioeconomic status [10,12,13]. One of these studies in particular looked at data for over 39,000 patients diagnosed with renal cell carcinoma from 1992 to 2007. This study found that 72.6% of White patients survived at least 5 years out from their diagnosis, while 68% of African Americans lived for at least 5 years. Another study using Department of Defense’s Automated Central Tumor Registry revealed a lack of racial difference in survival among RCC patients after adjusting for demographic, tumor, and treatment variables in the Cox model (adjusted HR = 1.08, 95% CI = 0.90–1.29) or stratified by age, sex, or tumor stage [16]. This study supports the claim that the lack of racial difference in survival among RCC patients may be related to equal access to health care.

Healthy People 2020 has identified the leading social determinants of health, such as personal, social, economic, lifestyle, and environmental factors, which are associated with individual health [17]. Black/African Americans show higher incidence and mortality rates of RCC compared to other ethnic groups. For example, scientific reports have shown that the prevalence of RCC has risen rapidly among African Americans compared to Caucasians [4,11,18]. These reports have also indicated that the differences in survival rate between African American and Caucasian patients are due to poor socioeconomic status, poor quality of life and living conditions, and potential limitations in treatment follow-up [11]. Although the reasons for this racial disparity have been addressed in the literature, little has been done to lower or reduce the gap observed in RCC health disparity. Several studies have reported the contributions of some physicians to racial/ethnic disparities in the transplantation of the kidney [1,19,20,21]. When compared with Caucasians, African American dialysis patients have more difficulty obtaining medical information, discussing with a medical professional about the possibility of receiving kidney from a family member, and obtaining proper health care [19].

### 3.2. Risk Factors of Renal Cell Carcinoma

**Figure 1:**
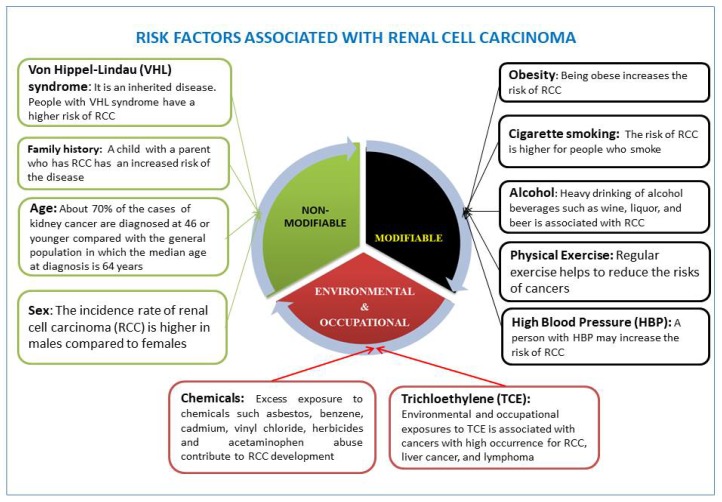
Leading risk factors affecting renal cell carcinoma (RCC) development are well recognized. These include nonmodifiable, modifiable (health behavioral and lifestyle), environmental, and occupational risk factors.

A person’s age and gender may increase his or her risk for RCC. Cigarette smoking, obesity, hypertension, and genetic diseases are the common risk factors that significantly contribute to the development of RCC [22,23,24]. Other risk factors related to lifestyle choice include heavy drinking of alcohol and high intake of fat and protein derived from animals [25,26,27]. Environmental and occupational factors also contribute largely to RCC development. Known risk factors associated with RCC are presented in Figure 1.

#### 3.2.1. Age and Sex Risk Factor in Renal Cell Carcinoma

About 75% of persons diagnosed with RCC are over the age of 60. RCC is rare in those under the age of 50. Incidence rates have increased in all age groups but this increase is mostly in persons over 75 years of age. Furthermore, mortality rates have mostly increased in persons over 75 years of age, validating that renal cancer is generally a disease of the elderly [28]. A study involving 600 cases of hereditary kidney cancer revealed that the median age is 37 years at diagnosis, but about 70% of the cases are diagnosed at 46 or younger [29], compared with the general population in which the median age at diagnosis is 64 years. One study found that patients with papillary RCC were significantly more likely to be older than those with clear cell, with ORs of 1.57 (95% CI 1.01–2.44) for those aged 60–69 years and 1.88 (95% CI 1.19–2.99) for those aged ≥70 years, compared with patients aged <50 years. Age at diagnosis did not appear to be differentially associated with either chromophobe or other RCC compared with clear cell [8].

The incidence rate of renal cell carcinoma (RCC) is higher in males compared with females. Men are two to three times more likely to be affected with RCC compared with women [1,30]. Men also have a lower survival rate compared with women. In 2012, there were approximately 337,860 new cases of kidney cancer (213,924 in males and 123,936 in females) and 143,406 deaths (90,802 in men and 52,604 in women) of kidney cancer patients [21]. Compared with clear cell RCC, patients with papillary RCC were less likely to be female (OR 0.60; 95% CI 0.43–0.83). Patients with chromophobe RCC were significantly more likely to be female compared with patients with clear cell RCC (OR 2.32; 95% CI 1.44–3.74) [8].

#### 3.2.2. Genetic Rick Factors in Renal Cell Carcinoma

The development of certain types of kidney cancers is inherited. Scientific data have identified genetic pathogenic variants that are the major cause of hereditary RCC risk in some prone families and these genetic pathogenic variants may contribute about 5%–8% of patients affected with RCC [31,32]. In 2014, Byler and Bratslavsky reported that hereditary RCC accounts for approximately 4% of cases and indicated a predilection towards early-onset, bilaterality, and multicentricity [33]. A study conducted by Karami et al. found that the first-degree relatives of hereditary kidney cancer patients have a high risk of developing RCC in both Caucasians and African Americans [34]. Furthermore, a large number of epidemiologic studies also revealed that family history is a major risk factor associated with RCC. For example, a relative risk is estimated to be about 2.5 for a brother or sister of a RCC patient [35,36]. A series of genome-wide association studies (GWAS) in the people of wealthy nations showed that loci mapped to endothelial PAS domain protein 1 gene (EPASI), encoding hypoxia inducible factor (HIF)-2α, on 2p21 (rs11894252 and rs7579899), a locus on 11q13.3 (rs7105934), a locus mapped to SCARB1, encoding the scavenger receptor class B, member 1 on 12q24.31 (rs4765623), a locus at a transcriptional enhancer of cyclin D1-encoding (CCND1) gene (rs71055934) at 11q13.3, two loci (rs718314 and rs1049380) in the inositol 1,4,5-triphosphate receptor, type 2 (ITPR2) gene on 12p11.23, and a locus (rs35252396) located at 8q24.21 were significantly associated with the susceptibility of RCC [37,38,39]. However, the above GWAS that have identified single nucleotide polymorphisms (SNPs) as a RCC risk factor in the people of wealthy nations could not be validated in similar studies performed in Chinese populations [40,41].

Hereditary RCC is predominantly caused by von Hippel–Lindau (VHL) syndrome, hereditary papillary renal cell carcinoma (HPRCC), and hereditary leiomyomatosis and RCC. VHL is an autosomal dominant condition with high penetrance; about half of the patients with VHL die from RCC. The VHL gene is a tumor suppressor and loss of the wild type allele is found in renal cysts and clear cell renal cancer from patients with VHL. Hereditary papillary renal cell carcinoma (HPRCC) is an autosomal dominant syndrome characterized by multifocal, bilateral type I papillary renal cell carcinomas [42]. Mutations of the MET gene on 7q31 have been linked HPRCC [43]. Hereditary leiomyomatosis and renal cell cancer (HLRCC) is an autosomal cancer susceptibility syndrome that develops cutaneous and uterine leiomyomas and renal cancer [44]. The most pathological type that is generally linked with HLRCC is papillary type 2 renal cancer and tends to have an early age of onset, be high grade, and aggressive [45]. Duct and clear cell cancers have also been associated with HLRCC [46,47,48]. Clear cell renal cancer is linked with VHL, chromosome 3 translocations, PTEN hamartomatous syndrome, and mutations in BAP1, as well as several of the genes encoding the proteins comprising the succinate dehydrogenase complex (SDHB/C/D) [49]. Birt–Hogg–Dubé syndrome (BHD) is a genetic condition associated with an increased risk of cancerous kidney tumors, especially chromophobe as well as clear cell renal cancer [50]. Tuberous sclerosis complex (TSC) is a genetic condition associated with RCC and linked to an increased risk of angiomyolipomas of the kidney [51]. Sequencing studies will continue to raise awareness of important causality and aggressive behavior related to the inherited genetics of RCC.

#### 3.2.3. High Blood Pressure/Hypertension Risk Factor in Renal Cell Carcinoma

High blood pressure is considered to be a major risk factor for renal cell carcinoma (RCC) development among African Americans and Caucasians, but African Americans suffer and die the most from this disease. A 2000 report revealed that African Americans have the highest incidence of hypertension at a younger age compared to other ethnic groups [52]. This highest prevalence of hypertension among African Americans at a younger age explains the highest incidence and mortality rates in this group and also because they are less likely to receive proper care [53]. In addition, African Americans often experience environmental stress in their community due the poor quality of life and living conditions which may increase RCC incidence in this group. A prospective study from eight European countries involving 296,638 subjects demonstrated that hypertension highly contributes to an increased risk of RCC [27]. Other studies showed a dose-response relationship between hypertension and RCC incidence when measuring blood pressure at the baseline clinic visits [54,55,56]. Compared with clear cell cases, chromophobe cases were significantly less likely than clear cell cases to have hypertension (OR 0.46; 95% CI 0.25–0.86) [8]. Obesity is a proven risk factor for RCC. When a study restricted regression models to those patients with available information on BMI and was further adjusted for BMI, patients with papillary, chromophobe, and other subtypes of RCC were all somewhat less likely to be obese than those with clear cell RCC, but these associations did not reach statistical significance [8]. Cardiovascular disease and diabetes are more prevalent in Hispanics [36] and type II diabetes is more prevalent among Native Americans [57]. All these disorders are associated with renal cancer development. Therefore, preventing hypertension-related risk factors will significantly reduce the incidence rate and improve the survival rate of RCC.

#### 3.2.4. Poor Diet and Alcohol Intake Risk Factor in Renal Cell Carcinoma

Regular consumption of vegetables and fruits may significantly reduce the prevalence rate of renal cell carcinoma (RCC). However, a poor diet is more likely to contribute to racial disparity in renal cancer. For example, diets with a high content of protein and dairy products have been associated with an increased risk of kidney cancer [58]. Diets rich in fruits and vegetables may provide a protective effect for RCC, but an unhealthy diet is considered a major risk factor in the pathogenesis of kidney cancer. Although there is an indication that the intake of protein and fat from animal origin is associated with a greater incidence of kidney cancer, other studies have shown inconclusive results [26,27,59]. Excess body weight or obesity is a significant public health issue in the United States and it is one of the major risk factors for kidney cancer [60,61]. It is documented that obesity accounts for over 40% of renal cell carcinoma (RCC) in the United States [62]. A 2003 study conducted on obesity and cancer risk among Caucasian and African American veterans found that the risk of RCC was significantly elevated among White men with or without hypertension, while the risk of RCC among African American men without hypertension was not elevated [63]. Moderate alcohol intake is linked to a reduction of RCC risk [64,65]. However, heavy drinking of alcoholic beverages such as wine, liquor, and beer is associated with RCC in males and females [34].

#### 3.2.5. Cigarette Smoking Risk Factor in Renal Cell Carcinoma

Cigarette smoking has been recognized as the major cause of human cancers and it can affect everyone (babies, children, and adults) at any stage of life [66]. Smoking is the best-established modifiable risk factor for RCC. About one in every five people who die in North America is attributed to the smoking of cigarettes, with an estimate of 440,000 deaths per year including 264,000 men and 178,000 women [66,67]. From this estimate, there are about 35,000 deaths resulting from second-hand smoke exposure. The risk of kidney cancer is not only greater in smokers than in nonsmokers, but is also increased with the amount and duration of smoking [68]. This increased risk has been attributed to several biologic mechanisms: smoking induces renal damage by toxic effects on the renal tubules and hemodynamic alterations including hypertension, endothelial cell dysfunction, and oxidative stress [69]. According to Cogliano and associates, cigarette smoking is classified as a risk for cancer in all anatomic areas of the renal upper tracts, including kidney, ureter, and renal pelvis [70]. A study of kidney cancer cases in the United Kingdom previously revealed that 29% of cases in males and 15% in females were associated with or caused by smoking [22]. A previous study by Ross and colleagues indicated that patients with a history of regular cigarette smoking have a 3.6-times chance of developing renal cancer compared to nonsmoking risk factors [71]. Another study found that smoking as a RCC risk factor applies to clear cell and papillary renal cell carcinoma but not the chromophobe subtype [72]. In addition, RCC patients were shown to have an increased amount of DNA damage in their peripheral blood lymphocytes induced by a tobacco-specific N-nitrosamine compared to control subjects [73].

#### 3.2.6. Environmental and Occupational Risk Factors in Renal Cell Carcinoma

There are many studies that have evaluated the risk of RCC in relation to environmental and occupational exposures in the United States. However, some scientific data remain inconclusive while others indicate that occupational exposure may be associated with the risk of RCC. Excess exposure to chemicals such asbestos, benzene, cadmium, vinyl chloride, and herbicides as well as acetaminophen abuse contribute to RCC development [74,75]. Several studies have reported that environmental and occupational exposures to trichloroethylene (TCE) is associated with cancers with high occurrence for RCC, liver cancer, and lymphoma [76,77]. TCE is a toxic solvent that can be found in degreasing agents, spot cleaning agents in dry cleaners, spray fixatives for arts and craft uses, and public drinking water supplies [76]. Using animal models, toxicological studies revealed that TCE-associated kidney damage occurs after bioactivation through the reductive metabolic pathway that is required prior to hepatic and renal glutathione S-transferase (GSH) conjugation and subsequent cleavage by renal cysteine conjugate β-lyase (CCBL1) to form cysteine *S*-conjugates, *S*-(1,2,dichlorovinyl-l-cytseine), and *S*-(1,2,2-trichlorovinyl l-cysteine) [78,79,80]. These metabolites are highly reactive and have been shown experimentally to form DNA adducts, strand breaks, bacterial mutagenicity, renal cell genotoxicity, and cytotoxicity [81,82]. A study from the National Health and Nutrition Examination Survey (NHANES) revealed that about 10% of people living in the United States have TCE in their body system which is detected in the blood [77]. According to the International Agency for Research on Cancer and the National Toxicology Program, TCE is considered as a probable human carcinogen [76,83], but its carcinogenic potential is inconsistent based on findings in epidemiologic studies.

## 4. Preventative Measures to Renal Cell Carcinoma

Although hereditary risk factors associated with renal cell carcinoma (RCC) are not modifiable, lifestyle and some environmental factors can be modified and prevented [84]. The best way to lower the prevalence rate of RCC is prevention. Interestingly, this will reduce the burden of RCC and lower the number of deaths caused by RCC in all ethnic groups. A healthy lifestyle can be achieved by keeping a normal body weight, eating well or consuming a high content of vegetable and fruits, lowering blood pressure, managing stress, maintaining enough physical activity, minimizing alcohol intake, quitting cigarette smoking, and reducing second-hand smoke exposure [85]. These therapeutic approaches to prevent RCC include lifestyle choices that are controllable and may reduce the risk of developing the disease. Below are some steps people can make to benefit their health and reduce their risk of developing RCC. Based on the scientific literature, it is evident that the environmental community where people live, work, perform daily activities, and physical exercise contribute significantly to health disparities.

### 4.1. Maintain a Healthy Diet

Lifestyle is responsible for large parts of cancer incidence in developed countries. Eating a healthy diet rich in vegetables and fruits, and intake of lean protein, nuts, whole grains, legumes, fish, and heart-healthy fats provides numerous human health benefits. The beneficial effects of a healthy diet may be mediated by favorable effects on blood pressure, glucose, and lipids [86,87]. Studies demonstrated that adopting a Mediterranean diet may reduce cancer and help the kidney to function well, while low adherence to it may lead to lower survival rates among patients with chronic kidney disease [88]. Other studies indicated that a plant-based diet influences the survival of renal cancer through its impact on metabolic acidosis and blood pressure [89]. Moreover, such a diet reduces urine parameters of kidney injury [90], decreases the production of potential uremic toxins through the alteration of gut flora [91], diminishes body weight, and improves cardiovascular outcomes [89,92]. A meta-analysis by Fouque et al. demonstrated a 39% reduction in the number of renal cancer deaths in patients on a very low protein diet [93]. Another meta-analysis of five trials including the Modification of Diet in Renal Disease study revealed a considerable reduction in renal failure risk and deaths (RR = 0.67) over 18–36 months of follow-up [94].

#### 4.1.1. Control Blood Pressure

Controlling blood pressure alone will not reduce the risk of developing RCC. A study performed by Colt et al. showed that well-controlled hypertension is still associated with a high risk of kidney cancer, with lower cancer rates among populations with poorly controlled blood pressure [95]. African American patients have a higher incidence of hypertension at a younger age than Caucasians and other ethnic groups [52]. This high incidence of hypertension among African Americans may explain the highest RCC incidence among this group and also provide insights into the presence of a more aggressive RCC in African Americans.

#### 4.1.2. Engage in Regular Physical Activity and Maintain a Healthy Weight

Scientific data indicated that regular exercise helps to reduce the risks of cancers through many mechanisms such as decreasing the levels of hormones that contribute to cancer development, improving the function of the immune system, and altering the metabolism of bile acids [96,97,98]. Research also indicates that regular physical exercise can lead to a reduction of weight which has a highly beneficial effect associated with cancer survival [99,100]. Regular physical exercise also contributes to a better quality of life such as emotional well-being and self-esteem [101].

#### 4.1.3. Quit Cigarette Smoking

Cigarette smoking is a major contributor to the high risk of developing kidney cancer. The risk drops once a person stops smoking. For example, a study that followed 845 patients with RCC found that 19.4% and 29.1% were current and former smokers, respectively. Also, 24.5% of these patients had advanced cancer. Smoking was consistently associated with advanced RCC in both univariate and multivariate analyses and cessation reversed the risk. One study revealed that patients who have a longer duration and exposure to smoking were associated with higher prevalence of advanced RCC, whereas duration cessation lowered the odds of advanced disease [102]. Since effective smoking cessation treatments exist for patients with cancer, the promotion of smoking cessation is warranted in patients with RCC who are current smokers or who are at an increased risk for relapse after quitting.

#### 4.1.4. Avoid Exposure to Toxic Chemicals

Avoiding environmental and occupational exposure to toxic chemicals such as arsenic, asbestos, cadmium, and organic solvents may reduce the incidence of RCC. Several environmental and occupational studies have reported that RCC is linked to some industrial employments such as dry cleaning, oil refining, metal working, and painting [103,104,105]. In addition, several toxic chemicals such as arsenic, cadmium, asbestos, gasoline, and solvents present in many occupational settings have been linked to an increased risk of RCC development [105,106].

### 4.2. Increase Awareness and Improve Minority Access to Healthcare

There is a significant racial disparity in survival for renal cell carcinoma (RCC) that exists between African Americans and Caucasians in the United States. This health disparity persists in particular among minority groups (African Americans in particular) and underserved populations. For example, African Americans and other minority Americans are less more likely to receive healthcare coverage through employment, and even if they have a job, they are still more likely to remain uninsured compared to Caucasians [107]. These disparities that African Americans and other minority Americans face in the United States are probably due to poor socioeconomic status, lack of education, and environmental stress. Poverty, lack of health insurance, barriers to screening, limited access to cancer treatment, poor quality of life, and stress associated with lower socioeconomic status are among the risk factors that contribute to these disparities. According to a study conducted by Berndt et al., the difference in survival rate between African Americans and Caucasians among RCC patients may be reduced simply by accounting for comorbidities and receiving nephrectomy among African American patients [107].

## 5. Conclusions

Although there is an immense quality improvement of renal cell carcinoma (RCC) among Caucasians, RCC remains a major public health issue among underrepresented minority (URM) groups. A likely contribution to the upward renal cancer incidence is the rising prevalence of obesity and hypertension. The impact of cigarette smoking should be monitored because the use of e-cigarettes is increasing even though cigarette smoking has declined. Furthermore, there is accumulating evidence to suggest that physical activity, alcohol intake, and occupational exposure to TCE may influence renal cell cancer risk. We must note that the relative contribution of each of these risk factors of RCC could vary according to the prevalence of other risk and protective factors, awareness, and effectiveness in the control of predisposing conditions. A small percentage of RCC cases are associated with familial cancer syndromes, genetic susceptibility, and its interplay with environmental exposures and is believed to play an etiologic role in the development of sporadic RCC. Lastly, most patients diagnosed with RCC are over the age of 60 are males. Despite the evidence of various associated risk factors, further research is needed to gain a greater understanding of the etiology of RCC. Therefore, the overall objective of this review paper was to discuss the major risk factors associated with RCC and highlight new therapeutic approaches for its control/prevention. New therapeutic approaches rather than surgical methods are needed to prevent and reduce RCC among African Americans, the most vulnerable URM group affected with the highest incidence and mortality rates of RCC. At the individual level, these new therapeutic solutions include keeping a normal body weight, eating well or consuming a high content of vegetable and fruits, lowering blood pressure, managing stress, maintaining enough physical activity, minimizing alcohol intake, quitting cigarette smoking, and reducing second-hand smoke exposure. To achieve best results in preventing RCC, physicians and healthcare policymakers should address the need for healthy lifestyle choices at the individual level, implement health care policies to improve societal inequities, facilitate minority access to healthcare programs, provide primary care services to the minority groups and uninsured populations, and emphasize the importance of public health in disease control and prevention.

## References

[1] Muglia V.F., Prando A. (2015). Renal cell carcinoma: Histological classification and correlation with imaging findings. Radiol. Bras..

[2] American Cancer Society (2018). Cancer Facts & Figures 2018.

[3] DeVita V.T. (2011). DeVita, Hellman, and Rosenberg’s Cancer: Principles and Practice of Oncology.

[4] Chow W.H., Devesa S.S., Warren J.L., Fraumeni J.F. (1999). Rising incidence of renal cell cancer in the United States. JAMA.

[5] US Cancer Statistics Working Group (2017). United States Cancer Statistics: 1999–2014 Incidence and Mortality Web-Based Report.

[6] Olshan A.F., Kuo T.M., Meyer A.M., Nielsen M.E., Purdue M.P., Rathmell W.K. (2013). Racial difference in histologic subtype of renal cell carcinoma. Cancer Med..

[7] Purdue M.P., Moore L.E., Merino M.J., Boffetta P., Colt J.S., Schwartz K.L., Bencko V., Davis F.G., Graubard B.I., Janout V. (2013). An investigation of risk factors for renal cell carcinoma by histologic subtype in two case-control studies. Int. J. Cancer.

[8] Lipworth L., Morgans A.K., Edwards T.L., Barocas D.A., Chang S.S., Herrell S.D., Penson D.F., Resnick M.J., Smith J.A., Clark P.E. (2016). Renal cell cancer histological subtype distribution differs by race and sex. BJU Int..

[9] Qi P., Tsivian M., Abern M.R., Passoni N.M., McGinley K.F., Polascik T.J. (2014). Clinicopathological characteristics and outcomes of surgically excised renal masses in African Americans. Urol. Oncol..

[10] Stafford H.S., Saltzstein S.L., Shimasaki S., Sanders C., Downs T.M., Sadler G.R. (2008). Racial/ethnic and gender disparities in renal cell carcinoma incidence and survival. J. Urol..

[11] Vaishampayan U.N., Do H., Hussain M., Schwartz K. (2003). Racial disparity in incidence patterns and outcome of kidney cancer. Urology.

[12] Chow W.H., Shuch B., Linehan W.M., Devesa S.S. (2013). Racial disparity in renal cell carcinoma patient survival according to demographic and clinical characteristics. Cancer.

[13] Berndt S.I., Carter H.B., Schoenberg M.P., Newschaffer C.J. (2007). Disparities in treatment and outcome for renal cell cancer among older black and white patients. J. Clin. Oncol..

[14] Eisenberg M.S., Leibovich B.C., Kim S.P. (2013). Racial disparity in renal cell carcinoma patient survival according to demographic and clinical characteristics. Cancer.

[15] Zini L., Perrotte P., Capitanio U., Jeldres C., Duclos A., Arjane P., Villers A., Montorsi F., Patard J.J., Karakiewicz P.I. (2009). Race affects access to nephrectomy but not survival in renal cell carcinoma. BJU Int..

[16] Lin J., Zahm S.H., Shriver C.D., Purdue M., McGlynn K.A., Zhu K. (2015). Survival among Black and White patients with renal cell carcinoma in an equal-access health care system. Cancer Causes Control.

[17] Koh H.K., Piotrowski J.J., Kumanyika S., Fielding J.E. (2011). Healthy people: A 2020 vision for the social determinants approach. Health Educ. Behav..

[18] Lipworth L., Tarone R.E., McLaughlin J.K. (2006). The epidemiology of renal cell carcinoma. J. Urol..

[19] Ayanian J.Z., Cleary P.D., Weissman J.S., Epstein A.M. (1999). The effect of patients’ preferences on racial differences in access to renal transplantation. N. Engl. J. Med..

[20] Hannan E.L., van Ryn M., Burke J., Stone D., Kumar D., Arani D., Pierce W., Rafii S., Sanborn T.A., Sharma S. (1999). Access to coronary artery bypass surgery by race/ethnicity and gender among patients who are appropriate for surgery. Med. Care.

[21] Ghoncheh M., Mirzaei M., Salehiniya H. (2015). Incidence and Mortality of Breast Cancer and their Relationship with the Human Development Index (HDI) in the World in 2012. Asian Pac. J. Cancer Prev..

[22] Parkin D.M. (2011). 2. Tobacco-attributable cancer burden in the UK in 2010. Br. J. Cancer.

[23] Perez-Gracia J.L., Prior C., Guillen-Grima F., Segura V., Gonzalez A., Panizo A., Melero I., Grande-Pulido E., Gurpide A., Gil-Bazo I. (2009). Identification of TNF-alpha and MMP-9 as potential baseline predictive serum markers of sunitinib activity in patients with renal cell carcinoma using a human cytokine array. Br. J. Cancer.

[24] Yu M.C., Mack T.M., Hanisch R., Cicioni C., Henderson B.E. (1986). Cigarette smoking, obesity, diuretic use, and coffee consumption as risk factors for renal cell carcinoma. J. Natl. Cancer Inst..

[25] Lee J.E., Hunter D.J., Spiegelman D., Adami H.O., Albanes D., Bernstein L., van den Brandt P.A., Buring J.E., Cho E., Folsom A.R. (2007). Alcohol intake and renal cell cancer in a pooled analysis of 12 prospective studies. J. Natl. Cancer Inst..

[26] Van Dijk B.A., Schouten L.J., Kiemeney L.A., Goldbohm R.A., van den Brandt P.A. (2005). Vegetable and fruit consumption and risk of renal cell carcinoma: Results from the Netherlands cohort study. Int. J. Cancer.

[27] Weikert S., Boeing H., Pischon T., Olsen A., Tjonneland A., Overvad K., Becker N., Linseisen J., Lahmann P.H., Arvaniti A. (2006). Fruits and vegetables and renal cell carcinoma: Findings from the European prospective investigation into cancer and nutrition (EPIC). Int. J. Cancer.

[28] Qayyum T., Oades G., Horgan P., Aitchison M., Edwards J. (2013). The epidemiology and risk factors for renal cancer. Curr. Urol..

[29] Schmid D., Leitzmann M.F. (2014). Association between physical activity and mortality among breast cancer and colorectal cancer survivors: A systematic review and meta-analysis. Ann. Oncol..

[30] Motzer R.J., Jonasch E., Agarwal N., Bhayani S., Bro W.P., Chang S.S., Choueiri T.K., Costello B.A., Derweesh I.H., Fishman M. (2017). Kidney Cancer, Version 2.2017, NCCN Clinical Practice Guidelines in Oncology. J. Natl. Compr. Cancer Netw..

[31] Gudbjartsson T., Jonasdottir T.J., Thoroddsen A., Einarsson G.V., Jonsdottir G.M., Kristjansson K., Hardarson S., Magnusson K., Gulcher J., Stefansson K. (2002). A population-based familial aggregation analysis indicates genetic contribution in a majority of renal cell carcinomas. Int. J. Cancer.

[32] Shuch B., Vourganti S., Ricketts C.J., Middleton L., Peterson J., Merino M.J., Metwalli A.R., Srinivasan R., Linehan W.M. (2014). Defining early-onset kidney cancer: Implications for germline and somatic mutation testing and clinical management. J. Clin. Oncol..

[33] Byler T.K., Bratslavsky G. (2014). Hereditary renal cell carcinoma: Genetics, clinical features, and surgical considerations. World J. Urol..

[34] Karami S., Daugherty S.E., Purdue M.P. (2015). A prospective study of alcohol consumption and renal cell carcinoma risk. Int. J. Cancer.

[35] Mucci L.A., Hjelmborg J.B., Harris J.R., Czene K., Havelick D.J., Scheike T., Graff R.E., Holst K., Moller S., Unger R.H. (2016). Nordic Twin Study of Cancer, C. Familial Risk and Heritability of Cancer Among Twins in Nordic Countries. JAMA.

[36] Teh B.T., Giraud S., Sari N.F., Hii S.I., Bergerat J.P., Larsson C., Limacher J.M., Nicol D. (1997). Familial non-VHL non-papillary clear-cell renal cancer. Lancet.

[37] Gudmundsson J., Sulem P., Gudbjartsson D.F., Masson G., Petursdottir V., Hardarson S., Gudjonsson S.A., Johannsdottir H., Helgadottir H.T., Stacey S.N. (2013). A common variant at 8q24.21 is associated with renal cell cancer. Nat. Commun..

[38] Purdue M.P., Johansson M., Zelenika D., Toro J.R., Scelo G., Moore L.E., Prokhortchouk E., Wu X., Kiemeney L.A., Gaborieau V. (2011). Genome-wide association study of renal cell carcinoma identifies two susceptibility loci on 2p21 and 11q13.3. Nat. Genet..

[39] Schodel J., Bardella C., Sciesielski L.K., Brown J.M., Pugh C.W., Buckle V., Tomlinson I.P., Ratcliffe P.J., Mole D.R. (2012). Common genetic variants at the 11q13.3 renal cancer susceptibility locus influence binding of HIF to an enhancer of cyclin D1 expression. Nat. Genet..

[40] Cao Q., Qin C., Ju X., Meng X., Wang M., Zhu J., Li P., Chen J., Zhang Z., Yin C. (2012). Chromosome 11q13.3 variant modifies renal cell cancer risk in a Chinese population. Mutagenesis.

[41] Su T., Han Y., Yu Y., Tan X., Li X., Hou J., Du Y., Shen J., Wang G., Ma L. (2013). A GWAS-identified susceptibility locus on chromosome 11q13.3 and its putative molecular target for prediction of postoperative prognosis of human renal cell carcinoma. Oncol. Lett..

[42] Zbar B., Glenn G., Lubensky I., Choyke P., Walther M.M., Magnusson G., Bergerheim U.S., Pettersson S., Amin M., Hurley K. (1995). Hereditary papillary renal cell carcinoma: Clinical studies in 10 families. J. Urol..

[43] Lindor N.M., Dechet C.B., Greene M.H., Jenkins R.B., Zincke M.T., Weaver A.L., Wilson M., Zincke H., Liu W. (2001). Papillary renal cell carcinoma: Analysis of germline mutations in the MET proto-oncogene in a clinic-based population. Genet. Test..

[44] Toro J.R., Nickerson M.L., Wei M.H., Warren M.B., Glenn G.M., Turner M.L., Stewart L., Duray P., Tourre O., Sharma N. (2003). Mutations in the fumarate hydratase gene cause hereditary leiomyomatosis and renal cell cancer in families in North America. Am. J. Hum. Genet..

[45] Merino M.J., Torres-Cabala C., Pinto P., Linehan W.M. (2007). The morphologic spectrum of kidney tumors in hereditary leiomyomatosis and renal cell carcinoma (HLRCC) syndrome. Am. J. Surg. Pathol..

[46] Alam N.A., Rowan A.J., Wortham N.C., Pollard P.J., Mitchell M., Tyrer J.P., Barclay E., Calonje E., Manek S., Adams S.J. (2003). Genetic and functional analyses of FH mutations in multiple cutaneous and uterine leiomyomatosis, hereditary leiomyomatosis and renal cancer, and fumarate hydratase deficiency. Hum. Mol. Genet..

[47] Grubb R.L., Franks M.E., Toro J., Middelton L., Choyke L., Fowler S., Torres-Cabala C., Glenn G.M., Choyke P., Merino M.J. (2007). Hereditary leiomyomatosis and renal cell cancer: A syndrome associated with an aggressive form of inherited renal cancer. J. Urol..

[48] Wei M.H., Toure O., Glenn G.M., Pithukpakorn M., Neckers L., Stolle C., Choyke P., Grubb R., Middelton L., Turner M.L. (2006). Novel mutations in FH and expansion of the spectrum of phenotypes expressed in families with hereditary leiomyomatosis and renal cell cancer. J. Med. Genet..

[49] Haas N.B., Nathanson K.L. (2014). Hereditary kidney cancer syndromes. Adv. Chronic Kidney Dis..

[50] Choueiri T.K., Vaishampayan U., Rosenberg J.E., Logan T.F., Harzstark A.L., Bukowski R.M., Rini B.I., Srinivas S., Stein M.N., Adams L.M. (2013). Phase II and biomarker study of the dual MET/VEGFR2 inhibitor foretinib in patients with papillary renal cell carcinoma. J. Clin. Oncol..

[51] Crino P.B., Nathanson K.L., Henske E.P. (2006). The tuberous sclerosis complex. N. Engl. J. Med..

[52] Vargas C.M., Ingram D.D., Gillum R.F. (2000). Incidence of hypertension and educational attainment: The NHANES I epidemiologic followup study. First National Health and Nutrition Examination Survey. Am. J. Epidemiol..

[53] Hollar D., Agatston A.S., Hennekens C.H. (2004). Hypertension: Trends, risks, drug therapies and clinical challenges in African Americans. Ethn. Dis..

[54] Macleod L.C., Hotaling J.M., Wright J.L., Davenport M.T., Gore J.L., Harper J., White E. (2013). Risk factors for renal cell carcinoma in the VITAL study. J. Urol..

[55] Setiawan V.W., Stram D.O., Nomura A.M., Kolonel L.N., Henderson B.E. (2007). Risk factors for renal cell cancer: The multiethnic cohort. Am. J. Epidemiol..

[56] Shen T., Shu X.O., Xiang Y.B., Li H.L., Cai H., Gao Y.T., Zheng W., Lipworth L. (2015). Association of hypertension and obesity with renal cell carcinoma risk: A report from the Shanghai Men’s and Women’s Health Studies. Cancer Causes Control.

[57] Egede L.E., Dagogo-Jack S. (2005). Epidemiology of type 2 diabetes: Focus on ethnic minorities. Med. Clin. N. Am..

[58] McCredie M., Ford J.M., Stewart J.H. (1988). Risk factors for cancer of the renal parenchyma. Int. J. Cancer.

[59] Lee J.E., Spiegelman D., Hunter D.J., Albanes D., Bernstein L., van den Brandt P.A., Buring J.E., Cho E., English D.R., Freudenheim J.L. (2008). Fat, protein, and meat consumption and renal cell cancer risk: A pooled analysis of 13 prospective studies. J. Natl. Cancer Inst..

[60] Luo J., Margolis K.L., Adami H.O., Lopez A.M., Lessin L., Ye W. (2007). Women’s Health Initiative, I. Body size, weight cycling, and risk of renal cell carcinoma among postmenopausal women: The Women’s Health Initiative (United States). Am. J. Epidemiol..

[61] McGuire B.B., Fitzpatrick J.M. (2011). BMI and the risk of renal cell carcinoma. Curr. Opin. Urol..

[62] Calle E.E., Kaaks R. (2004). Overweight, obesity and cancer: Epidemiological evidence and proposed mechanisms. Nat. Rev. Cancer.

[63] Samanic C., Gridley G., Chow W.H., Lubin J., Hoover R.N., Fraumeni J.F. (2004). Obesity and cancer risk among white and black United States veterans. Cancer Causes Control.

[64] Bellocco R., Pasquali E., Rota M., Bagnardi V., Tramacere I., Scotti L., Pelucchi C., Boffetta P., Corrao G., La Vecchia C. (2012). Alcohol drinking and risk of renal cell carcinoma: Results of a meta-analysis. Ann. Oncol..

[65] Song D.Y., Song S., Song Y., Lee J.E. (2012). Alcohol intake and renal cell cancer risk: A meta-analysis. Br. J. Cancer.

[66] Centers for Disease Control and Prevention (1993). Cigarette smoking-attributable mortality and years of potential life lost—United States, 1990. MMWR Morb. Mortal. Wkly. Rep..

[67] Boring C.C., Squires T.S., Tong T., Heath C.W. (1993). From the Centers for Disease Control and Prevention. Mortality trends for selected smoking-related cancers and breast cancer—United States, 1950–1990. JAMA.

[68] Hunt J.D., van der Hel O.L., McMillan G.P., Boffetta P., Brennan P. (2005). Renal cell carcinoma in relation to cigarette smoking: Meta-analysis of 24 studies. Int. J. Cancer.

[69] Orth S.R. (2002). Cigarette smoking: An important renal risk factor—Far beyond carcinogenesis. Tob. Induc. Dis..

[70] Cogliano V.J., Baan R., Straif K., Grosse Y., Lauby-Secretan B., El Ghissassi F., Bouvard V., Benbrahim-Tallaa L., Guha N., Freeman C. (2011). Preventable exposures associated with human cancers. J. Natl. Cancer Inst..

[71] Ross R.K., Paganini-Hill A., Landolph J., Gerkins V., Henderson B.E. (1989). Analgesics, cigarette smoking, and other risk factors for cancer of the renal pelvis and ureter. Cancer Res..

[72] Patel N.H., Attwood K.M., Hanzly M., Creighton T.T., Mehedint D.C., Schwaab T., Kauffman E.C. (2015). Comparative Analysis of Smoking as a Risk Factor among Renal Cell Carcinoma Histological Subtypes. J. Urol..

[73] Clague J., Shao L., Lin J., Chang S., Zhu Y., Wang W., Wood C.G., Wu X. (2009). Sensitivity to NNKOAc is associated with renal cancer risk. Carcinogenesis.

[74] Hu J., Mao Y., White K. (2002). Renal cell carcinoma and occupational exposure to chemicals in Canada. Occup. Med..

[75] Pastore A.L., Palleschi G., Silvestri L., Moschese D., Ricci S., Petrozza V., Carbone A., Di Carlo A. (2015). Serum and urine biomarkers for human renal cell carcinoma. Dis. Markers.

[76] International Agency for Research on Cancer (1999). Re-evaluation of some organic chemicals, hydrazine and hydrogen peroxide. Proceedings of the IARC Working Group on the Evaluation of Carcinogenic Risks to Humans.

[77] Wu C., Schaum J. (2000). Exposure assessment of trichloroethylene. Environ. Health Perspect..

[78] Anders M.W., Dekant W., Vamvakas S. (1992). Glutathione-dependent toxicity. Xenobiotica.

[79] Dekant W., Vamvakas S., Anders M.W. (1990). Biosynthesis, bioactivation, and mutagenicity of S-conjugates. Toxicol. Lett..

[80] Lash L.H., Putt D.A., Parker J.C. (2006). Metabolism and tissue distribution of orally administered trichloroethylene in male and female rats: Identification of glutathione- and cytochrome P-450-derived metabolites in liver, kidney, blood, and urine. J. Toxicol. Environ. Health A.

[81] Monks T.J., Anders M.W., Dekant W., Stevens J.L., Lau S.S., van Bladeren P.J. (1990). Glutathione conjugate mediated toxicities. Toxicol. Appl. Pharmacol..

[82] Muller M., Birner G., Dekant W. (1998). Reactivity of haloketenes and halothioketenes with nucleobases: Chemical characterization of reaction products. Chem. Res. Toxicol..

[83] National Toxicology Program (2011). Report on Carcinogens.

[84] US Department of Health and Human Services (2014). The Health Consequences of Smoking-50 Years of Progress: A Report of the Surgeon General.

[85] National Heart, Lung, and Blood Institute (2014). Risk Assessment Tool for Estimating Your 10-Year Risk of Having a Heart Attack. NIH.

[86] Belin R.J., Greenland P., Allison M., Martin L., Shikany J.M., Larson J., Tinker L., Howard B.V., Lloyd-Jones D., Van Horn L. (2011). Diet quality and the risk of cardiovascular disease: The Women’s Health Initiative (WHI). Am. J. Clin. Nutr..

[87] Weinstein S.J., Vogt T.M., Gerrior S.A. (2004). Healthy Eating Index scores are associated with blood nutrient concentrations in the third National Health and Nutrition Examination Survey. J. Am. Diet. Assoc..

[88] Castro-Quezada I., Roman-Vinas B., Serra-Majem L. (2014). The Mediterranean diet and nutritional adequacy: A review. Nutrients.

[89] Goraya N., Wesson D.E. (2015). Dietary interventions to improve outcomes in chronic kidney disease. Curr. Opin. Nephrol. Hypertens..

[90] Goraya N., Simoni J., Jo C.H., Wesson D.E. (2013). A comparison of treating metabolic acidosis in CKD stage 4 hypertensive kidney disease with fruits and vegetables or sodium bicarbonate. Clin. J. Am. Soc. Nephrol..

[91] Evenepoel P., Meijers B.K., Bammens B.R., Verbeke K. (2009). Uremic toxins originating from colonic microbial metabolism. Kidney Int. Suppl..

[92] Estruch R., Ros E., Salas-Salvado J., Covas M.I., Corella D., Aros F., Gomez-Gracia E., Ruiz-Gutierrez V., Fiol M., Lapetra J. (2013). Primary prevention of cardiovascular disease with a Mediterranean diet. N. Engl. J. Med..

[93] Brunori G., Viola B.F., Parrinello G., De Biase V., Como G., Franco V., Garibotto G., Zubani R., Cancarini G.C. (2007). Efficacy and safety of a very-low-protein diet when postponing dialysis in the elderly: A prospective randomized multicenter controlled study. Am. J. Kidney Dis..

[94] Pedrini M.T., Levey A.S., Lau J., Chalmers T.C., Wang P.H. (1996). The effect of dietary protein restriction on the progression of diabetic and nondiabetic renal diseases: A meta-analysis. Ann. Intern. Med..

[95] Colt J.S., Schwartz K., Graubard B.I., Davis F., Ruterbusch J., DiGaetano R., Purdue M., Rothman N., Wacholder S., Chow W.H. (2011). Hypertension and risk of renal cell carcinoma among white and black Americans. Epidemiology.

[96] Bernstein H., Bernstein C., Payne C.M., Dvorakova K., Garewal H. (2005). Bile acids as carcinogens in human gastrointestinal cancers. Mutat. Res..

[97] Wertheim B.C., Martinez M.E., Ashbeck E.L., Roe D.J., Jacobs E.T., Alberts D.S., Thompson P.A. (2009). Physical activity as a determinant of fecal bile acid levels. Cancer Epidemiol. Biomarkers Prev..

[98] Winzer B.M., Whiteman D.C., Reeves M.M., Paratz J.D. (2011). Physical activity and cancer prevention: A systematic review of clinical trials. Cancer Causes Control.

[99] Rock C.L., Doyle C., Demark-Wahnefried W., Meyerhardt J., Courneya K.S., Schwartz A.L., Bandera E.V., Hamilton K.K., Grant B., McCullough M. (2012). Nutrition and physical activity guidelines for cancer survivors. CA Cancer J. Clin..

[100] Speck R.M., Courneya K.S., Masse L.C., Duval S., Schmitz K.H. (2010). An update of controlled physical activity trials in cancer survivors: A systematic review and meta-analysis. J. Cancer Surviv..

[101] Mishra S.I., Scherer R.W., Geigle P.M., Berlanstein D.R., Topaloglu O., Gotay C.C., Snyder C. (2012). Exercise interventions on health-related quality of life for cancer survivors. Cochrane Database Syst. Rev..

[102] Tsivian M., Moreira D.M., Caso J.R., Mouraviev V., Polascik T.J. (2011). Cigarette smoking is associated with advanced renal cell carcinoma. J. Clin. Oncol..

[103] Asal N.R., Geyer J.R., Risser D.R., Lee E.T., Kadamani S., Cherng N. (1988). Risk factors in renal cell carcinoma. II. Medical history, occupation, multivariate analysis, and conclusions. Cancer Detect. Prev..

[104] Auperin A., Benhamou S., Ory-Paoletti C., Flamant R. (1994). Occupational risk factors for renal cell carcinoma: A case-control study. Occup. Environ. Med..

[105] Pesch B., Haerting J., Ranft U., Klimpel A., Oelschlagel B., Schill W. (2000). Occupational risk factors for renal cell carcinoma: Agent-specific results from a case-control study in Germany. MURC Study Group. Multicenter urothelial and renal cancer study. Int. J. Epidemiol..

[106] Parent M.E., Hua Y., Siemiatycki J. (2000). Occupational risk factors for renal cell carcinoma in Montreal. Am. J. Ind. Med..

[107] KFF (2011). Kaiser Commission on Medicaid and the Uninsured.

